# Job satisfaction among public health professionals working in public sector: a cross sectional study from Pakistan

**DOI:** 10.1186/1478-4491-11-2

**Published:** 2013-01-09

**Authors:** Ramesh Kumar, Jamil Ahmed, Babar Tasneem Shaikh, Rehan Hafeez, Assad Hafeez

**Affiliations:** 1 Department of Health Systems & Policy, Health Services Academy, Government of Pakistan, Chak Shahzad, Park Road, Opposite NIH, Islamabad 44000, Pakistan

**Keywords:** Job satisfaction, Public sector, Health professionals, Job descriptions

## Abstract

**Background:**

Job satisfaction largely determines the productivity and efficiency of human resource for health. It literally depicts the extent to which professionals like or dislike their jobs. Job satisfaction is said to be linked with the employee’s work environment, job responsibilities and powers and time pressure; the determinants which affect employee’s organizational commitment and consequently the quality of services. The objective of the study was to determine the level of and factors influencing job satisfaction among public health professionals in the public sector.

**Methods:**

This was a cross sectional study conducted in Islamabad, Pakistan. Sample size was universal including 73 public health professionals, with postgraduate qualifications and working in government departments of Islamabad. A validated structured questionnaire was used to collect data from April to October 2011.

**Results:**

Overall satisfaction rate was 41% only, while 45% were somewhat satisfied and 14% of professionals highly dissatisfied with their jobs. For those who were not satisfied, working environment, job description and time pressure were the major causes. Other factors influencing the level of satisfaction were low salaries, lack of training opportunities, improper supervision and inadequate financial rewards.

**Conclusion:**

Our study documented a relatively low level of overall satisfaction among workers in public sector health care organizations. Considering the factors responsible for this state of affairs, urgent and concrete strategies must be developed to address the concerns of public health professionals as they represent a highly sensitive domain of health system of Pakistan. Improving the overall work environment, review of job descriptions and better remuneration might bring about a positive change.

## Background

Efficiency and productivity of human resources depends upon many factors, and job satisfaction is one of the most important. Human resource in any institution is the most valuable asset and it works as an engine to provide a sustainable service delivery. Pakistan is already facing a serious shortfall in terms of human resources for health. Research suggests that where the private health sector is relatively well organized and has better working conditions, the workers within the public sector face numerous issues that grossly affect the level of job satisfaction [1]. Job satisfaction is known to be a multifaceted phenomenon that entails an individual’s feelings towards his/her job [2]. In clinical sciences, doctors’ satisfaction plays a great role in their performance and therefore is reflected as satisfaction and compliance among their patients. Similarly, public health professionals’ satisfaction in their jobs would determine the quality of the service delivery for their respective communities [3,4]. Organizational factors and poor working conditions have also been strongly associated with job dissatisfaction, while the social aspects of a job have been found to be a strong predictor of job satisfaction [5]. The phenomenon of satisfaction has also been inversely associated with absenteeism, turnover in an organization, level of stress and eventual exhaustion [6,7]. Job satisfaction in workers is a very important factor that influences productivity, as well as the quality of work within an organization. This intricate phenomenon is an attitude towards one’s job that has an impact not only on the personnel’s motivation, but also on career, health and relationships with co-workers [8-10]. Moreover, low salaries, lack of fringe benefits, job insecurity, nepotism, political influences and improper career development structure are some of the important factors that either hinder qualified public health professionals from joining the public sector or increase the turnover rate [11].

Although many studies have been done to address the question of the level of job satisfaction among public health doctors, very few have been conducted in Pakistan, especially in the recent past. Currently employed public health professionals also seem to be less satisfied due to many unidentified factors and hence there is a constant threat of attrition. This study attempted to document the level of job satisfaction, and determine the factors influencing job satisfaction among public health professionals working in the government sector.

## Methods

This was a descriptive cross-sectional study conducted from April to October 2011. Public health professionals of various cadres (management, administration, academia, research) were enrolled from Islamabad, the capital territory. The Manual for Minnesota Satisfaction Questionnaire^a^, a validated tool for such surveys, was used for this study. Pre-testing was done with an aim to check its format, language, sequence, comprehension of the questions among the participants and duration of one interview. The final data collection tool was slightly modified and then adapted by adding few open-ended questions. The reliability and validity of the tool was again checked using Cronbach’s alpha test and was found acceptable.

The study was approved by the Institutional Review Board at the parent organization of the principal investigator (PI). The study participants were chosen on the basis of their basic qualification Bachelor of Medicine and Bachelor of Surgery (MBBS), work place (essentially government organization/department), postgraduate qualification in public health, and number of years of experience. A total of 73 participants were found to be eligible for the study and they were all enrolled to answer a self-administered questionnaire. All participants were assured of confidentiality and anonymity, when they gave consent to participate. The response rate was 100%. The Statistical Package for Social Sciences (SPSS) version 17 was used to analyze the quantitative data which included both descriptive and inferential statistics.

## Results

Among the respondents, 70% were male and 30% female. More than half (56%) of the participants worked in senior-level public health managerial positions and had experience of more than 10 years; 62% of participants were working as permanent and 38% as contractual employees. About one third (36%) had a monthly income between PkRs40,000 to 60,000 while only 15% had a monthly income more than PkRs80,000. Two thirds of respondents had a Master of Public Health (MPH) as their highest qualification and 27% had a Master of Science (MSc) while only 7% were PhD holders. There was no significant association found between socio-demographic characteristics and factors affecting the job satisfaction.

**Figure 1:**
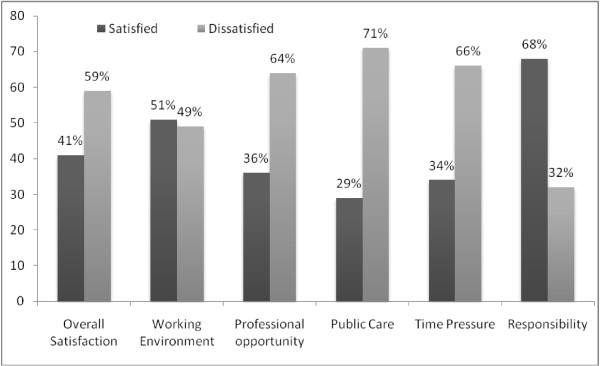
Overall level of satisfaction among professionals of the public sector.

Figure 1 shows that overall, 59% participants were dissatisfied with their job (this includes 14% who were absolutely dissatisfied and 45% who were somewhat satisfied). Only half of the respondents were satisfied with their working environment, whereas 68% were satisfied with their responsibilities. A majority (71%) was dissatisfied with the quality of services they provided to their clients in their jobs; 66% of the participants were dissatisfied due to the irrelevant tasks assigned and lack of decision-making in their job. About two-thirds of public health professionals were not satisfied with the professional opportunities, resources and the work schedule.

Table 1 shows the inferential statistics with overall job satisfaction as an outcome variable. The outcome variable was computed by merging the questions and then putting them on a Likert scale. Respondents who were dissatisfied with their jobs overall were also significantly dissatisfied with the environment at their workplace (*P* < 0.0005), with their responsibilities at their organizations (*P* < 0.0005) and the time pressure they faced at work (*P* < 0.009).

**Table 1:** Factors associated with the overall level of job satisfaction

**Variable**	**Mean score (SD)**		**Total score**	**P-Value**
	**Satisfied**	**Dissatisfied**		
Working environment	21.6 (5.1)	28 (5.7)	50	< 0.0005
Responsibility	4.6 (1.5)	7.6 (2.7)	15	< 0.0005
Time pressure	4.6 (1.4)	5.3 (1.1)	10	0.009
Public Health Care	8.4 (2.6)	9.2 (2.3)	15	0.180
Professional opportunity	15.1 (3.2)	15.1 (2.4)	30	0.523

Pearson correlation was used to measure the relationship between general satisfaction and each individual job satisfaction dimension. Overall, general satisfaction and all the individual components of job satisfaction had a positive relationship with each other at a low to moderate level. However, there was no relationship between overall satisfaction and professional opportunity. Overall job satisfaction had a significant positive moderate association with working environment (*r* = 0.65), responsibility (*r* = 0.65), time pressure (*r* = 0.30), and public health care (*r* = 0.47), all at a *P*-value < 0.01 (Table 2).

**Table 2:** Pearson correlations between overall level of job satisfaction and other factors

	**Overall satisfaction**	**Working environment**	**Responsibility**	**Public care**	**Time pressure**	**Professional opportunity**
Overall satisfaction	1	0.653**	0.658**		0.301**	
Working environment	0.653**	1	0.517**	0.477**	0.314**	
Responsibility	0.658**	0.517**	1	0.405**		
Public care		0.477**	0.405**	1		
Time pressure	0.301**	0.314**			1	
Professional opportunity						1

*Correlation is significant at the 0.01 level (2-tailed).

In order to capture more contextual and relevant to our own public health system, some of the other factors documented by virtue of some open ended questions were as below:

1. Dignity

2. Low salary

3. Lack of trainings opportunities and career structure

4. Inadequate supervision

5. Insufficient cooperation and professional support

6. Too little financial rewards and freedom to work

## Discussion

Our study showed that a majority of the participants were not satisfied with their job (14% highly dissatisfied and 45% somewhat satisfied). Overall dissatisfaction among public health professionals at public sector is a cause for concern, given that the factor of job satisfaction could have implications for the overall efficiency, effectiveness and sustainability of the Pakistani health system. Working conditions at public sector health organizations do not meet the values and aspirations of public health professionals. Pakistan has a mix of public and private health care system, in which the public sector is usually under-resourced and yet serves the majority of population. Government spending on health is only 0.5% of GDP or US$6.4 per capita which is certainly not enough to keep the staff morale high [12].

People respond unfavorably to restrictive work environments, therefore, it is imperative for organizations to create incentivized environment that enable the employees to achieve the highest level of job satisfaction [13]. Professional autonomy motivates employees to perform at their best and show commitment to the organization, enhancing work conditions to support the organization’s mission and thus impacting on job satisfaction. The conditions under which jobs are performed can have as much impact on people’s effectiveness, comfort and safety as the intrinsic details of the task itself [14]. A recent study conducted in Tanzania also reported the poor job satisfaction in their health system due to lack of job description, poor rewards system, discouraging working environment and weak communications in the staff [15].

In our study, public health professionals reported low satisfaction with professional development opportunities, recognition, poor salaries and benefits, not being involved in decision making, doing a lot of irrelevant task and having sufficient time pressure. Employees’ needs and motivators vary so it is important to understand what motivates them to perform. In the current study, variables such as the irrelevant responsibilities, improper working environment and time pressure were seen to have a significant influence on the job satisfaction. A study conducted in Sweden proved that the liberty of decision making for health manager is very important for the organizational betterment and apt performance. According to their results, organizational support in this regard improves work satisfaction and mental energy, and decreases work related exhaustion among physicians. It diminishes the turnover rate among physicians [16]. Otherwise, irrelevant tasks assigned and non availability of a clear work plan could be the main factors contributing to mismanagement of working hours [17].

Dissatisfaction with the work is directly reflected by the poor output of the organizations [18]. Results of another study confirmed the contribution of emotional demands to prediction of emotional exhaustion and their effects on job satisfaction levels [19]. Numerous studies conducted among healthcare professionals point to the importance of the interpersonal relationships in job satisfaction, and that it is likely to increase the client safety, improved quality of care and greater client satisfaction [20].

Our study showed that the majority of the public health respondents were dissatisfied with the professional and development opportunities they availed during their professional life. Literature shows human resource in health sector needs continuous training and refreshers [21]. Training increases the self confidence and self esteem of health professionals and improves the quality of care that would significantly elevate the morale of health professionals in the organization [22].

The present study also indicated that the respondents were dissatisfied with their income, lack of resources and minimal involvement in decision-making. These findings are similar to those of study conducted in Lithuania, where the professionals were dissatisfied with the degree of personal autonomy in clinical decision-making, the amount of time spent with patients and their salaries [23].

This study found no significant differences between permanent and contractual employees on various determinants of job satisfaction. This contrasts with the Turkish study, which found that those working with public sector were not satisfied and have a low motivation due to their nature of contract [24].

Since it was a self-administered questionnaire, it is therefore possible that the respondents might have over- or under-reported their level of satisfaction. The findings of the study may not be generalized to public health professionals working in the private sector in the country.

## Conclusion

Satisfaction with one’s job can affect not only motivation at work but also career decisions, relationships with others and personal health. Those who work in a profession that is extremely demanding and sometimes unpredictable can be susceptible to feelings of uncertainty and reduced job satisfaction. Job satisfaction of public health workers is also an essential part of ensuring high quality care in the programs they are employed in. We envisage to share the results of this study with the relevant authorities in the study area as well as with the provincial health departments to interpret the same in their own context and act upon accordingly. The message to the policy makers will not only point towards the actions to be taken but it will attempt to sensitize them on the subject of satisfaction of the public health workforce and its implications. Interventions should be carried out to increase levels of job satisfaction among public health professionals at public sector. It is imperative to reinforce the relevant human resources polices, improve working conditions and revise the compensation scales. It is recommended that employees’ job description be redesigned to have a scope of enrichment and interest. Continuous service evaluations and the monitoring of job satisfaction can be useful to determine various aspects of the services that necessitate improvement. Involving the health professionals in a cooperative team approach will allow deliberations on ways to improve the level of job satisfaction. A conducive environment in line with the aspirations of the public health professionals is likely to increase job satisfaction and consequently to have a positive effect on individual, organizational and quality of health care services in today’s evolving health system of Pakistan.

## Endnote

^a^University of Minnesota. Department of Psychology. http://www.psych.umn.edu/psylabs/vpr/msqinf.htm

## Competing interests

Authors declare that there are no competing interests.

## Authors’ contribution

RK conceived and designed the study and collected the data, JA analyzed the data and analyzed the results. BTS, RH and AH helped in preparing, editing and finalizing the manuscript for publication.
